# Effectiveness of hand hygiene practices in preventing influenza virus infection in the community setting: A systematic review

**DOI:** 10.14745/ccdr.v45i01a02

**Published:** 2019-01-03

**Authors:** K Moncion, K Young, M Tunis, S Rempel, R Stirling, L Zhao

**Affiliations:** 1 Centre for Immunization and Respiratory Infectious Diseases, Public Health Agency of Canada, Ottawa, ON

**Keywords:** community, hand hygiene, hand sanitizer, handwashing, influenza infection, influenza transmission, systematic review

## Abstract

**Background:**

Hand hygiene is known to be an effective infection prevention and control measure in health care settings. However, the effectiveness of hand hygiene practices in preventing influenza infection and transmission in the community setting is not clear.

**Objective:**

To identify, review and synthesize available evidence on the effectiveness of hand hygiene in preventing laboratory-confirmed or possible influenza infection and transmission in the community setting.

**Methods:**

A systematic review protocol was established prior to conducting the review. Three electronic databases (MEDLINE, Embase and the Cochrane Library) were searched to identify relevant studies. Two reviewers independently screened the titles, abstracts and full-texts of studies retrieved from the database searches for potential eligibility. Data extraction and quality assessment of included studies were performed by a single reviewer and validated by a second reviewer. Included studies were synthesized and analyzed narratively.

**Results:**

A total of 16 studies were included for review. Studies were of low methodological quality and there was high variability in study design, setting, context and outcome measures. Nine studies evaluated the effectiveness of hand hygiene interventions or practices in preventing laboratory-confirmed or possible influenza infection in the community setting; six studies showed a significant difference, three studies did not. Seven studies assessed the effectiveness of hand hygiene practices in preventing laboratory-confirmed or possible influenza transmission in the community setting; two studies found a significant difference and five studies did not.

**Conclusion:**

The effectiveness of hand hygiene against influenza virus infection and transmission in the community setting is difficult to determine based on the available evidence. In light of its proven effectiveness in other settings, there is no compelling evidence to stop using good hand hygiene practice to reduce the risk of influenza infection and transmission in the community setting.

## Introduction

Hand hygiene is a commonly recommended infection prevention and control measure to reduce the risk of influenza infection and transmission in health care and community settings. Routine hand hygiene protocols that indicate the use of soap and running water to wash hands (1) and/or alcohol-based hand sanitizers to rub hands (1,2) are effective at physically removing influenza virus from human hands.

Hand hygiene practices have been found to be effective in reducing infection and transmission of healthcare-associated pathogens in the health care setting (3); in reducing non-pathogen-specific gastrointestinal and respiratory illnesses in the community setting (4,5); and for disinfection, removal of contaminants and reduction of the incidence of hospital-acquired infections in the health care setting (3).

Less frequently studied has been the degree of protection against influenza virus infection and transmission afforded by hand hygiene practices in the community setting. An initial scoping search of the literature identified two systematic reviews that came to different conclusions. A review of randomized controlled trials found that hand hygiene as a co-intervention with facemask use in the community setting was efficacious against laboratory-confirmed influenza infection or influenza-like illness, but hand hygiene alone was not (6). Another review of intervention trials and observational studies found evidence of a reduction in influenza infection with hand hygiene interventions in schools, but no effect on secondary transmission of influenza in households in the community that had already experienced an index case (7).

A systematic review was undertaken to identify, review and synthesize the latest evidence on the effectiveness of hand hygiene as an intervention in preventing laboratory-confirmed or possible influenza infection and transmission in the community setting. The term “possible influenza infection” was defined as non-laboratory-confirmed cases, including influenza-like illness or an acute respiratory illness.

## Methods

The systematic review parameters, search strategy and analysis plan were established prior to the conduct of the review. Hand hygiene was defined as handwashing, hand antisepsis and actions taken to maintain healthy hands and fingernails (8). The search strategy (**Appendix 1**) was developed in collaboration with a research librarian. MEDLINE, Embase and the Cochrane Library electronic databases were searched from inception until June 5, 2017 using search terms for influenza and hand hygiene. Searches were restricted to articles published in English or French.

Studies were included for review if they met the following criteria:

- They were conducted in a community setting, which is defined as a non-health care, open setting without confinement and without special care for the participants (e.g., school, workplace, household) (6)
- They were observational studies that assessed hand hygiene as an exposure of interest (e.g., observed or reported hand hygiene practice) or clinical trials that could include combinations of education, promotion and provision of products to do with hand hygiene, but assessed a hand hygiene intervention that could be reasonably expected to exert an independent influence
- They assessed the impact of hand hygiene on:
  - laboratory-confirmed or possible influenza infection or
  - laboratory-confirmed or possible influenza transmission

Studies were excluded if they met one or more of the following criteria:

- They were conducted in the health care setting only
- They assessed a multicomponent intervention for which hand hygiene could not be reasonably expected to exert an independent influence
- They were not clinical research studies (e.g., literature reviews, editorials, opinion pieces or news stories, or non-human or in vitro studies)

Study selection was completed independently by two reviewers. Reference lists of included studies and relevant secondary research articles retrieved through the search were also searched to identify relevant publications. One reviewer (KM) performed data extraction and quality appraisal and a second reviewer performed validation (LZ). Data were extracted on study design, population, setting, hand hygiene intervention (i.e., from clinical trials) or practice (i.e., from observational studies) and outcomes of interest. Study quality was assessed using the Cochrane Collaboration Risk of Bias Tool for randomized controlled trials (RCTs) (9) and the Effective Public Health Practice Project Quality Assessment Tool for observational designs (10). Disagreements between the two reviewers were resolved by discussion and reaching a consensus.

Narrative data synthesis and analysis were planned to summarize the direction, size and statistical significance of reported effect estimates for various study-defined outcomes and to explore overall patterns in the data extracted from included studies. If possible, meta-analyses were planned to assess the association of hand hygiene with influenza outcomes by income level of country of study, study design, setting, intervention evaluated and outcome assessed.

## Results

**Figure 1:**
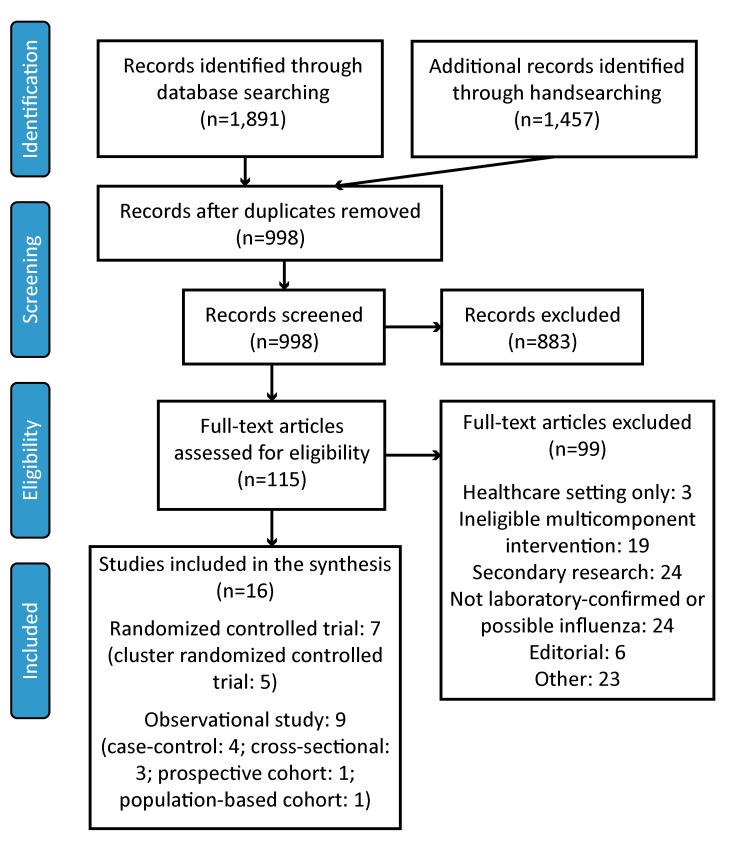
Flow diagram of the study selection process Abbreviation: n, number

After database searching, handsearching and removal of duplicates, 998 records remained. After screening, 115 records were identified for full-text review. When all inclusion and exclusion criteria were applied, 16 studies—seven RCTs and nine observational studies—were available for review. **Figure 1** summarizes the study selection process.

RCTs assessed using the Cochrane Collaboration Risk of Bias Tool were all found to be at a high risk of bias (11–17). Observational studies assessed using the Effective Public Health Practice Project Quality Assessment Tool found seven of nine observational studies as weak in quality (18–24) and two as moderate in quality (25,26). The reviewers made a post-hoc decision to not perform a meta-analysis as the limited number of included studies were not adequate for grouping by the study characteristics of interest.

### RCTs on hand hygiene interventions

Of the seven included RCTs, six assessed the provision of hand sanitizer or soap with instructions on their use (11–14,16,17). One RCT delivered an internet-based intervention educating and promoting handwashing without provision of any hand sanitizer or soap to participants (15). None of these RCTs reported the instructions or education given to participants on handwashing or hand antisepsis in sufficient detail to compare the appropriateness of these interventions to best practices.

### Observational studies on hand hygiene practices

Of the nine included observational studies, four collected self-reported handwashing frequency (21,24–26). Of the remaining five studies, one study dichotomized observed handwashing behaviour as observed or not observed (18) and one as frequent or infrequent (19). These studies did not specify or report the use of handwashing criteria in estimating handwashing frequency or counting handwashing events. Two studies assessed self-reported quality of hand hygiene practice, that is, good or poor (20), and optimal or suboptimal (23), and of these, one defined optimal hand hygiene practice according to published best practices (20). Another study collected self-reported information on adoption of various non-pharmaceutical interventions, including washing hands more often and hand sanitizer use (22).

### Hand hygiene and influenza infection

Nine studies evaluated the effectiveness of hand hygiene interventions or practices in preventing laboratory-confirmed or possible influenza infection in the community setting, including two RCTs (13,15), one cohort study (25), three case-control studies (19,21,26) and three cross-sectional studies (18,20,23).

Study findings were mixed; six of nine studies found that some form of hand hygiene intervention or practice reduced laboratory-confirmed (21,26) or possible (15,18,20,23) influenza infection, while three studies found hand hygiene to be not statistically significantly associated with a decrease in influenza infection (13,19,25). For the two RCTs, one found a significant association between handwashing and decreased risk of influenza-like illness (15) and the other found no effect on self-reported clinically diagnosed influenza for a workplace hand sanitizer intervention (13). For the observational studies, which relied on self-reported (20,21,23,25,26) or observed (18,19) hand hygiene practice, most found statistically significantly lower likelihood of possible infection (18,20,21,23,26). The limited number of heterogeneous studies did not allow for more granular qualitative analysis of findings. The results are summarized in **Table 1**.

**Table 1:** Summary of evidence related to the effectiveness of hand hygiene practices in preventing laboratory-confirmed or possible influenza infection in the community setting

Study	Sample size (n)	Hand hygiene intervention or reported practice/ control intervention	Main outcome measure	Relevant key findings
Randomized controlled trial				
Hubner et al., 2010 (13)	134 (intervention: 67; control: 67)	Instruction to use an alcohol-based hand disinfectant at least five times daily only at work, with disinfectant provided Control: No instruction or disinfectant provided	Self-report of clinically diagnosed influenza	Intervention and control groups did not differ in likelihood of clinically diagnosed influenza (OR: 1.02, 95% CI: 0.20–5.23)
Little et al., 2015 (15)	20,066 (intervention: 10,040; control: 10,026)	Access to web-based intervention providing information about the importance of influenza and the role of HW Control: No access to the web-based intervention	ILI	Participants in the intervention group had a decreased risk of reported ILI in the past four months (aRR: 0.80, 95% CI: 0.72–0.92) and in the past month (aRR: 0.85, 95% CI: 0.77–0.94) compared to the control group
Cohort study				
Merk et al., 2004 (25)	4,365	Self-reported HW frequency	Self-reported ILI and ARI	Adults who washed their hands ≥5 times per day and those who washed their hands two to four times per day did not statistically significantly differ in incidence of ILI (aRR: 1.10–1.48) and ARI (aRR: 1.08–1.22)
Case-control study				
Doshi et al., 2015 (19)	486 (case: 145; control: 341)	Observed household level HW behaviour (frequent/infrequent)	Laboratory-confirmed influenza	Household level HW with soap and water was not statistically significantly associated with laboratory-confirmed influenza (aOR: 1.06, 95% CI: 0.90–1.24)
Liu et al., 2016 (21)	200 (case: 100; control: 100)	Self-reported HW frequency	Laboratory-confirmed influenza	HW statistically significantly decreased the likelihood of laboratory-confirmed influenza (by 54% per unit increase in HW score; aOR: 0.46, 95% CI: 0.29–0.74)
Torner et al., 2015 (26)	478 (case: 239; control: 239)	Self-reported HW frequency	Laboratory-confirmed influenza	Children who reported washing their hands ≥5 times a day had a statistically significantly lower likelihood of laboratory-confirmed influenza compared to those who did not (aOR: 0.62, 95% CI: 0.39–0.99). The use of alcohol-based HS (aOR: 1.54, 95% CI: 0.8–2.66) and HW after touching contaminated surfaces (aOR: 0.62, 95% CI: 0.29–1.31) were not statistically significantly associated with laboratory-confirmed influenza
Cross-sectional study				
Adesanya et al., 2016 (18)	28,596	Observed HW behaviour (observed/not observed)	Parent-reported ARI	Children who were observed to not wash their hands had an increased likelihood of having ARI symptoms compared to children who were observed to wash their hands (aOR: 1.66, 95% CI: 1.33–2.07)
Hashim et al., 2016 (20)	468	Self-reported hand hygiene practice (good/poor)	Self-reported respiratory illness (ILI and non-ILI)	Hajj pilgrims with self-reported good hand hygiene practice had a statistically significantly lower likelihood of developing respiratory illness compared to those who did not report good hand hygiene practice (OR: 0.41, 95% CI: 0.20–0.85)
Wu et al., 2016 (23)	13,003	Self-reported HW or HS use (optimal/suboptimal)	Self-reported ILI	Optimal hand hygiene (definition not provided) was found to be statistically significantly associated with a lower likelihood of reporting ILI (OR: 0.87, 95% CI: 0.80–0.94)

Abbreviations: ARI, acute respiratory illness; aOR, adjusted odds ratio; aRR, adjusted rate ratio; CI, confidence interval; HS, hand sanitizer; HW, handwashing; ILI, influenza-like illness; OR, odds ratio; ≥, superior or equal to

### Hand hygiene and influenza transmission

Seven studies assessed the effectiveness of hand hygiene practices in preventing laboratory-confirmed or possible influenza transmission in the community setting, including five RCTs (11,12,14,16,17), one cohort study (22), and one case-control study (24). A majority of these studies assessed influenza transmission in the community setting by estimating secondary attack rates (SARs) at the household level (e.g., the proportion of susceptible individuals who became ill) for laboratory-confirmed or possible influenza (11,12,14,16,17).

Five of seven studies did not find a statistically significant association between hand hygiene intervention or practice and influenza transmission (11,12,14,16,22). An RCT found a statistically significant difference in SARs for influenza-like illness across handwashing, handwashing and facemask, and control interventions (0.17, 0.18 and 0.09, respectively), but not in SARs for laboratory-confirmed influenza (17). A case-control study found that handwashing at least three times per day was statistically significantly associated with reduced likelihood of household transmission of pandemic influenza A (H1N1) (24).

In four of five cluster RCTs conducted at the household level, hand hygiene intervention was implemented after the identification of the index case (11,12,16,17). Two of these four studies assessed a subgroup of households where the intervention was implemented within a defined period after the onset of symptoms in the index case (e.g., less than 36 or 48 hours); one of the two studies did not find a statistically significant difference between hand hygiene and control groups (12) while the other study found mixed results, depending on influenza type and determination of influenza (17). Four of five cluster RCTs did not find statistically significant differences in SARs for laboratory-confirmed or possible influenza between hand hygiene and control groups (11,12,14,16) and one found mixed results depending on outcome (17). The results are summarized in **Table 2**.

**Table 2:** Summary of evidence related to the effectiveness of hand hygiene practices in preventing laboratory-confirmed or possible influenza transmission in the community setting

Study	Sample size (n)	Hand hygiene intervention or reported practice/ control intervention	Main outcome measure	Relevant key findings
Randomized controlled trial				
Cowling et al., 2008 (11)	198 households (hand hygiene: 36; FM: 35; control: 127)	Hand hygiene intervention: Same education as control intervention plus hand hygiene education (potential efficacy of proper hand hygiene in reducing transmission and instructions) and provision of HS and soap FM intervention: Same education as control intervention plus FM education and provision of FMs to each household member Control: Healthy diet and lifestyle education with respect to illness prevention for household contacts and symptom alleviation for the index subject	SARs for clinical (three definitions) or laboratory-confirmed influenza	SARs for clinical and laboratory-confirmed influenza did not statistically significantly differ across the intervention arms. The likelihood of secondary infection in a household contact was statistically similar between the hand hygiene intervention and control groups for clinical (OR: 0.80–0.86) and laboratory-confirmed (OR: 1.07) influenza
Cowling et al., 2009 (12)	407 households (hand hygiene: 136; hand hygiene and FM: 137; control: 134)	Hand hygiene intervention: Same education as control intervention plus hand hygiene education (potential efficacy of proper hand hygiene in reducing transmission and instructions) and provision of HS and soap Hand hygiene and FM intervention: Same education as control and hand hygiene interventions plus FM education and provision of FM to each household member Control: Healthy diet and lifestyle education with respect to illness prevention for household contacts and symptom alleviation for the index subject	SARs for clinical (two definitions) and laboratory-confirmed influenza	SAR for clinical and laboratory-confirmed secondary cases did not statistically significantly differ across the intervention arms. The likelihood of secondary infection in a household contact was statistically similar comparing the hand hygiene intervention group for clinical (OR: 0.92–0.81) and laboratory-confirmed (OR: 0.57) influenza and the hand hygiene plus FM intervention group for clinical (OR: 1.25–1.68) and laboratory-confirmed (OR: 0.77) influenza to the control group
Larson et al., 2010 (14)	509 households (HS: 169; HS and FM: 166; control: 174)	HS intervention: Educational materials and HS to be carried by individual household members to work or school HS and FM intervention: Educational materials, HS, FMs and instructions on FM use Control: Educational materials regarding the prevention and treatment of URI and influenza	ILI and laboratory-confirmed influenza SARs for URI, ILI and laboratory-confirmed influenza	Intervention and control groups did not differ in rates of ILI or laboratory-confirmed influenza SARs for URI, ILI and laboratory-confirmed influenza were similar across interventions (HS: 0.144; HS and FM: 0.124; and control: 0.137) Restricting outcomes to ILI and laboratory-confirmed influenza, SARs were similar across interventions (HS: 0.020; HS and FM: 0.018; and control: 0.023)
Ram et al., 2015 (16)	377 households (HW: 193; control: 184)	HW education and promotion and provision of HW station with soap and water after illness onset in the index case Control: Standard practice	SARs for ILI and laboratory-confirmed influenza	SAR ratios for ILI (1.24, 95% CI: 0.93–1.65) and laboratory-confirmed influenza (2.40, 95% CI: 0.68–8.47) comparing intervention to control households were not statistically significant.
Simmerman et al., 2011 (17)	465 households (HW: 155; HW and FM: 155; control: 155)	HW intervention: HW education and soap dispenser HW and FM intervention: HW education, soap dispenser and FMs Control: Nutritional, physical activity and smoking cessation education	SARs for ILI and laboratory-confirmed influenza	SARs for ILI were statistically significantly different across interventions (HW: 0.17; HW and FM: 0.18; and comparison: 0.09; *p*=0.01). However, SARs for laboratory-confirmed influenza were not statistically significantly different across interventions (HW: 0.23; HW and FM: 0.23; and control: 0.19; *p*=0.63). Other analyses for influenza transmission found similar associations for ILI and laboratory-confirmed influenza outcomes comparing intervention and control groups
Cohort study				
Loustalot et al., 2011 (22)	2,030	Self- and proxy-reported household-level hand hygiene behaviour (HW frequency and HS use)	Reported ILI in household	Households with at least one reported case of ILI did not statistically significantly differ in reported HW frequency (*p*=0.34) or HS use (*p*=0.37) compared to households without ILI
Case-control study				
Zhang et al., 2013 (24)	162 households (case household: 54; control household: 108)	Self-reported HW frequency	Laboratory-confirmed influenza	HW ≥3 times per day was statistically significantly associated with reduced likelihood of household transmission of pandemic influenza A (H1N1) (OR: 0.71, 95% CI: 0.48–0.94)

Abbreviations: CI, confidence interval; FM, facemask; HS, hand sanitizer; HW, handwashing; ILI, influenza-like illness; OR, odds ratio; SAR, secondary attack rate; URI, upper respiratory infection; ≥, superior or equal to

## Discussion

The present systematic review identified 16 studies that assessed the impact of hand hygiene intervention or practice on influenza infection or transmission in the community setting. Two-thirds of studies suggested hand hygiene practices may help prevent influenza infection. Most studies that looked at influenza transmission, however, had non-statistically significant results. Most studies had design elements associated with the potential for bias. The studies were too heterogeneous in design for meta-analysis. Our findings were similar to the two other systematic reviews conducted on this issue despite methodological differences in study selection. Whereas we found both positive and negative studies, the Wong et al. review (6) found that hand hygiene intervention alone was not efficacious against laboratory-confirmed influenza and the Warren-Gash et al. review (7) found some evidence of influenza risk reduction with hand hygiene intervention, depending on the community setting. Warren-Gash et al. also found no evidence of effectiveness of hand hygiene on secondary transmission of influenza in households that had already experienced an index case (7).

### Limitations

There are a number of important limitations to consider when interpreting the findings of this review. In general, the majority of studies investigated outcomes that were not specific to influenza virus infection, but were influenza-like illness and acute respiratory illness, which could be caused by other respiratory viruses. Findings from lower income settings (e.g., rural Bangladesh) may not be generalizable to high-income settings and vice versa. Moreover, in controlled clinical trials conducted in high-income settings, there may already be high baseline levels of hand hygiene practice rendering intervention and control groups more similar irrespective of hand hygiene intervention. The effectiveness of hand hygiene interventions is dependent on mode of influenza transmission and may be attenuated when the mode of transmission is not through contact. The present review restricted its scope to hand hygiene interventions independent of other public health measures; therefore, these interventions may not be reflective of real-world, multicomponent public health measures. Finally, a search of the grey literature was not undertaken, so some studies may have been missed.

There were also limitations inherent to both types of study. Some of the included RCTs lacked statistical power (11,13,14). None of the included RCTs presented information on hand hygiene interventions in sufficient detail to allow the comparison of these interventions to best practices. Possible non-compliance with the intervention and contamination of control participants may underestimate possible effects of hand hygiene. Adoption of effective hand hygiene practice may take longer than the intervention period of a clinical trial. For RCTs investigating influenza transmission in households with an index case, it is possible that the hand hygiene intervention was implemented too late in the course of illness of the index case to be effective in preventing intra-household transmission. In household studies, direct and indirect protection conferred by hand hygiene practice for more susceptible individuals (e.g., children) cannot be readily assessed due to a lack of information on hand hygiene practice collected at the individual level.

For the included observational studies, where hand hygiene practices were either self-reported or observed, measurement of hand hygiene practice may be influenced by response bias (e.g., social desirability bias), recall bias or the observer effect (27). Although most observational studies collected exposure data on self-reported handwashing frequency, these studies did not specify or report the use of criteria for counting handwashing events; therefore, optimal and suboptimal hand hygiene practices cannot be differentiated in the overall reported handwashing frequency. Observational studies may also be susceptible to residual confounding, selection bias and other biases that may further complicate the interpretation of findings. Although the cross-sectional studies included for review found statistically significant results (18,20,23), the cross-sectional design cannot determine whether the reported hand hygiene behaviour preceded influenza illness.

### Implications and next steps

These numerous limitations of the existing body of evidence highlight the difficulties of conducting research on this topic in the community setting for both experimental and observational designs (6,7,28). Hand hygiene is a non-invasive, non-pharmaceutical intervention without adequate comparator interventions (29). There are also challenges in conducting RCTs with appropriate sample sizes to establish the relative importance of hand hygiene (30). In the community setting, it is also difficult to implement interventions and assess outcomes.

In light of the robust body of evidence on the benefits of hand hygiene practices with respect to general infectious disease prevention and control (31), the mixed results and limitations of current studies, there is no compelling evidence to stop using good hand hygiene practice to reduce the risk of influenza infection and transmission in the community. Hand hygiene practices are non-invasive and have broad applicability as an infection prevention and control intervention with no demonstrated evidence of harm.

Further research would help to clarify whether, and under what circumstances, hand hygiene interventions in the community are effective in preventing influenza infection and transmission.

### Conclusion

Available evidence on the effectiveness of hand hygiene practices in preventing influenza infection and transmission in the community is inconsistent and insufficient in both quality and quantity. However, in light of its efficacy in general infectious disease prevention and control, there is no compelling evidence to stop using good hand hygiene practice to reduce the risk of influenza infection and transmission in the community.

## Appendix 1 Electronic database search strategy and results

**Table:**

Set #	Searches	Results
MEDLINE		
1	hand hygiene/ or hand disinfection/	5,680
2	(hand? adj3 (hygien* or wash* or disinfect* or sanitiz* or antiseptic* or steriliz* or decontaminat* or clean*)).tw.	7,433
3	handwash*.tw.	1,661
4	1 or 2 or 3	10,550
5	exp residence characteristics/ or exp schools/ or workplace/ or exp "Non-Medical Public and Private Facilities"/	280,888
6	(communit* or domicile? or domestic or residential or neighborhood? or household? or home? or family or families or school* or college? or universit* or "education* setting*" or student? or daycare? or childcare or workplace? or workspace? or worksite? or employee? or "public setting?" or "non healthcare setting*" or "non health care setting*").tw.	2,148,929
7	((work or job or public) adj3 (setting? or location? or site? or place?)).tw.	15,472
8	5 or 6 or 7	2,296,190
9	influenza, human/ or exp influenzavirus a/ or exp influenzavirus b/	63,179
10	(influenza* or flu or h1n# or h2n# or h3n# or h5n# or h6n# or h7n# or h9n# or h10n#).tw.	110,315
11	common cold/ or respiratory tract infections/ or rhinitis/ or sinusitis/ or fever/ or cough/ or pharyngitis/ or sneezing/ or myalgia/ or headache/ or vomiting/ or diarrhea/	201,878
12	("common cold" or "respiratory infection*" or "respiratory virus*" or "respiratory tract infection*" or "respiratory illness*" or fever* or cough* or "sore throat" or "runny nose" or "nasal congestion" or sneezing or malaise* or myalgia or headache* or "muscle ache*" or vomit* or diarrhea or diarrhoea).tw.	419,905
13	9 or 10 or 11 or 12	616,262
14	4 and 8 and 13	717
15	limit 14 to (english or french)	674
16	15 and "Editorial" [Publication Type]	2
17	15 and "Newspaper Article" [Publication Type]	1
18	15 not (16 or 17)	671
19	hand hygiene/ or hand disinfection/	5,680
20	(hand? adj3 (hygien* or wash* or disinfect* or sanitiz* or antiseptic* or steriliz* or decontaminat* or clean*)).tw.	7,433
21	handwash*.tw.	1,661
22	19 or 20 or 21	10,550
23	influenza, human/ or exp influenzavirus a/ or exp influenzavirus b/	63,179
24	(influenza* or flu or h1n# or h2n# or h3n# or h5n# or h6n# or h7n# or h9n# or h10n#).tw.	110,315
25	common cold/ or respiratory tract infections/ or rhinitis/ or sinusitis/ or fever/ or cough/ or pharyngitis/ or sneezing/ or myalgia/ or headache/ or vomiting/ or diarrhea/	201,878
26	("common cold" or "respiratory infection*" or "respiratory virus*" or "respiratory tract infection*" or "respiratory illness*" or fever* or cough* or "sore throat" or "runny nose" or "nasal congestion" or sneezing or malaise* or myalgia or headache* or "muscle ache*" or vomit* or diarrhea or diarrhoea).tw.	419,905
27	23 or 24 or 25 or 26	616,262
28	22 and 27	1,349
29	limit 28 to (english or french)	1,249
30	29 and "Editorial" [Publication Type]	15
31	29 and "Newspaper Article" [Publication Type]	3
32	29 and "Comment" [Publication Type]	32
33	29 not (30 or 31 or 32)	1,203
34	33 not 18	538
Embase		
1	hand washing/ or hand disinfection/	11,298
2	(hand? adj3 (hygien* or wash* or disinfect* or sanitiz* or antiseptic* or steriliz* or decontaminat* or clean*)).tw.	10,307
3	handwash*.tw.	1,863
4	1 or 2 or 3	16,007
5	community/ or community living/ or household/ or home/ or exp school/ or workplace/ or building/	456,912
6	(communit* or domicile? or domestic or residential or neighborhood? or household? or home? or family or families or school* or college? or universit* or "education* setting*" or student? or daycare? or childcare or workplace? or workspace? or worksite? or employee? or "public setting?" or "non healthcare setting*" or "non health care setting*").tw.	2,757,553
7	((work or job or public) adj3 (setting? or location? or site? or place?)).tw.	19,320
8	5 or 6 or 7	2,899,020
9	exp influenza/ or exp influenza virus/	88,859
10	(influenza* or flu or h1n# or h2n# or h3n# or h5n# or h6n# or h7n# or h9n# or h10n#).tw.	126,819
11	common cold/ or respiratory tract infection/ or fever/ or rhinitis/ or sinusitis/ or coughing/ or sore throat/ or rhinorrhea/ or nose obstruction/ or pharyngitis/ or sneezing/ or myalgia/ or headache/ or vomiting/ or diarrhea/	737,993
12	("common cold" or "respiratory infection*" or "respiratory virus*" or "respiratory tract infection*" or "respiratory illness*" or fever* or cough* or "sore throat" or "runny nose" or "nasal congestion" or sneezing or malaise* or myalgia or headache* or "muscle ache*" or vomit* or diarrhea or diarrhoea).tw.	562,610
13	9 or 10 or 11 or 12	1,087,580
14	4 and 8 and 13	1,092
15	limit 14 to (english or french)	1,041
16	15 and "Editorial" [Publication Type]	6
17	15 not 16	1,035
18	hand washing/ or hand disinfection/	11,298
19	(hand? adj3 (hygien* or wash* or disinfect* or sanitiz* or antiseptic* or steriliz* or decontaminat* or clean*)).tw.	10,307
20	handwash*.tw.	1,863
21	18 or 19 or 20	16,007
22	exp influenza/ or exp influenza virus/	88,859
23	(influenza* or flu or h1n# or h2n# or h3n# or h5n# or h6n# or h7n# or h9n# or h10n#).tw.	126,819
24	common cold/ or respiratory tract infection/ or fever/ or rhinitis/ or sinusitis/ or coughing/ or sore throat/ or rhinorrhea/ or nose obstruction/ or pharyngitis/ or sneezing/ or myalgia/ or headache/ or vomiting/ or diarrhea/	737,993
25	("common cold" or "respiratory infection*" or "respiratory virus*" or "respiratory tract infection*" or "respiratory illness*" or fever* or cough* or "sore throat" or "runny nose" or "nasal congestion" or sneezing or malaise* or myalgia or headache* or "muscle ache*" or vomit* or diarrhea or diarrhoea).tw.	562,610
26	22 or 23 or 24 or 25	1,087,580
27	21 and 26	2,512
28	limit 27 to (english or french)	2,370
29	28 and "Editorial" [Publication Type]	68
30	28 not 29	2,302
31	30 not 17	1,267
Cochrane Library		
1	[mh ^"hand hygiene"] or [mh ^"hand disinfection"]	363
2	(hand? near/3 (hygien* or wash* or disinfect* or sanitiz* or antiseptic* or steriliz* or decontaminat* or clean*)):ti,ab,kw	154
3	handwash*:ti,ab,kw	217
4	1 or 2 or 3	544
5	[mh ^"residence characteristics"] or [mh schools] or [mh ^workplace] or [mh "Non-Medical Public and Private Facilities"]	3,578
6	(communit* or domicile? or domestic or residential or neighborhood? or household? or home? or family or families or school* or college? or universit* or (education* next setting*) or student? or daycare? or childcare or workplace? or workspace? or worksite? or employee? or (public next setting?) or "non healthcare setting" or "non health care setting" or "non healthcare settings" or "non health care settings"):ti,ab,kw	101,164
7	((work or job or public) near/3 (setting? or location? or site? or place?)):ti,ab,kw	248
8	5 or 6 or 7	101,724
9	[mh ^"influenza, human"] or [mh "influenzavirus a"] or [mh "influenzavirus b"]	1,830
10	(influenza* or flu or h1n? or h2n? or h3n? or h5n? or h6n? or h7n? or h9n? or h10n?):ti,ab,kw	7,611
11	[mh ^"common cold"] or [mh ^"respiratory tract infections"] or [mh ^rhinitis] or [mh ^sinusitis] or [mh ^fever] or [mh ^cough] or [mh ^pharyngitis] or [mh ^sneezing] or [mh ^myalgia] or [mh ^headache] or [mh ^vomiting] or [mh ^diarrhea]	13,353
12	("common cold" or (respiratory next infection*) or (respiratory next virus*) or (respiratory next tract next infection*) or (respiratory next illness*) or fever* or cough* or "sore throat" or "runny nose" or "nasal congestion" or sneezing or malaise* or myalgia or headache* or (muscle next ache*) or vomit* or diarrhea or diarrhoea):ti,ab,kw	77,363
13	9 or 10 or 11 or 12	82,910
14	4 and 8 and 13	86
15	[mh ^"hand hygiene"] or [mh ^"hand disinfection"]	363
16	(hand? near/3 (hygien* or wash* or disinfect* or sanitiz* or antiseptic* or steriliz* or decontaminat* or clean*)):ti,ab,kw	154
17	handwash*:ti,ab,kw	217
18	15 or 16 or 17	544
19	[mh ^"influenza, human"] or [mh "influenzavirus a"] or [mh "influenzavirus b"]	1,830
20	(influenza* or flu or h1n? or h2n? or h3n? or h5n? or h6n? or h7n? or h9n? or h10n?):ti,ab,kw	7,611
21	[mh ^"common cold"] or [mh ^"respiratory tract infections"] or [mh ^rhinitis] or [mh ^sinusitis] or [mh ^fever] or [mh ^cough] or [mh ^pharyngitis] or [mh ^sneezing] or [mh ^myalgia] or [mh ^headache] or [mh ^vomiting] or [mh ^diarrhea]	13,353
22	("common cold" or (respiratory next infection*) or (respiratory next virus*) or (respiratory next tract next infection*) or (respiratory next illness*) or fever* or cough* or "sore throat" or "runny nose" or "nasal congestion" or sneezing or malaise* or myalgia or headache* or (muscle next ache*) or vomit* or diarrhea or diarrhoea):ti,ab,kw	77,363
23	19 or 20 or 21 or 22	82,910
24	18 and 23	127
25	24 not 14	41
